# Advancing enhanced recovery after surgery protocols for pediatric laparoscopic-assisted small intestinal malformation repair

**DOI:** 10.1186/s12887-026-06516-z

**Published:** 2026-01-20

**Authors:** Kai Zhu, Hengfei Gao, Yaqi Chen, Ying Zhou, Mingyun Hong, Yilin Su

**Affiliations:** 1 Department of Pediatric Surgery, The First Affiliated Hospital of USTC, Division of Life Sciences and Medicine, University of Science and Technology of China, Hefei, 230001 Anhui Province China; 2 Reproductive Medicine Center, Hefei Maternal and Child Health Hospital, Hefei, 230032 Anhui Province China

**Keywords:** Enhanced recovery after surgery, Meckel's diverticulum, Intestinal duplication, Small intestinal malformation, Perioperative care

## Abstract

**Purpose:**

This study aimed to evaluate the safety and efficacy of enhanced recovery after surgery (ERAS) protocols in pediatric patients undergoing laparoscopic-assisted resection for Meckel’s diverticulum (MD) or intestinal duplication (ID).

**Methods:**

A retrospective cohort analysis was conducted on 96 pediatric patients who underwent laparoscopic-assisted resection for MD or ID at our institution between January 2017 and July 2025, covering the periods pre- and post-ERAS implementation. Patients were stratified into two groups: the ERAS group (*n* = 49), managed per ERAS protocols, and the traditional (TRAD) group (*n* = 47), receiving conventional perioperative care. Demographic characteristics, perioperative outcomes, and laboratory parameters were systematically compared between groups.

**Results:**

All procedures were performed by a consistent surgical team, with no significant differences in baseline characteristics between groups (all *P* > 0.05). The ERAS group exhibited superior outcomes: (1) Recovery: shorter median postoperative length of stay (LOS) (7.00 vs. 9.00 days, *P* < 0.001) and consistently lower FLACC pain scores at 2–48 h postoperatively (*P* < 0.001); (2) Laboratory markers: comparable preoperative values (all *P* > 0.05), but higher glucose levels at anesthesia induction (*P* < 0.001) and favorable postoperative laboratory profiles (lower C-reactive protein [CRP], neutrophil [NEUT] count, and glucose; higher prealbumin; all *P* < 0.05); (3) Clinical benefits: reduced urinary catheter utilization and duration (both *P* < 0.05), accelerated achievement of recovery milestones (mobility, flatus, oral intake, total enteral nutrition initiation, and intravenous infusion cessation; all *P* < 0.001), and lower healthcare costs (*P* < 0.001) without compromising safety (complication and readmission rates, both *P* > 0.05). ERAS-related advantages were more pronounced, with a non-significant trend toward higher parental satisfaction (*P* = 0.444).

**Conclusions:**

ERAS protocols safely optimize recovery in pediatric patients undergoing laparoscopic-assisted resection for small intestinal malformations (MD/ID) without adversely affecting clinical outcomes.

**Supplementary Information:**

The online version contains supplementary material available at 10.1186/s12887-026-06516-z.

Meckel’s diverticulum (MD) and intestinal duplication (ID) are the two most common congenital small intestinal anomalies in children [1]. MD results from failed regression of the omphalomesenteric duct during fetal development; this true diverticulum, composed of normal ileal layers, typically locates within 100 cm of the ileocecal valve on the antimesenteric border [2]. Most cases require surgical intervention due to complications including bleeding, obstruction, inflammation, or intussusception [3]. ID presents as cystic or tubular structures adherent to the mesenteric border of the bowel, sharing a muscular wall with the adjacent intestine; the ileum and ileocecal region are most frequently involved [4]. Complete surgical resection is mandatory to prevent complications such as bleeding, volvulus, necrosis, and malignancy [5]. Given that duplicated segments share a blood supply with normal bowel, adjacent intestinal tissue may need to be resected concurrently. Laparoscopic- assisted resection has emerged as the preferred surgical approach for both conditions in children, reflecting advances in minimally invasive techniques [6].

The enhanced recovery after surgery (ERAS) protocol, developed by Professor Henrik Kehlet, is an evidence-based multimodal strategy designed to optimize perioperative care by mitigating surgical stress and accelerating recovery [7]. While well-established in adult populations [8, 9], ERAS implementation in pediatrics has been slower due to unique challenges: smaller blood volume, immature thermoregulation, developing immunity, higher nutritional requirements, and limited communication capabilities [10], which necessitate pediatric-specific adaptations.

Based on clinical evidence [11, 12], we developed a tailored ERAS protocol for pediatric laparoscopic resection of MD or ID. This study systematically compared outcomes between the ERAS protocol and traditional perioperative care (TRAD) to evaluate the safety and efficacy of ERAS in the surgical management of these small intestinal malformations.

## Methods

### Study population

This retrospective cohort study analyzed pediatric patients with small bowel malformations (MD or ID) who were treated at The First Affiliated Hospital of the University of Science and Technology of China between January 2017 and July 2025.

Inclusion criteria: (1) Laparoscopically confirmed MD or ID with pathological verification; (2) Hemodynamically stable (hemoglobin > 80 g/L) without indications for emergency surgery due to acute complications (e.g., complete intestinal obstruction, intestinal bleeding with progressive Hb decline despite conservative treatment, or bowel perforation with septic shock); (3) Only concurrent appendectomy permitted; (4) Immediate postoperative transfer to the pediatric surgical ward; (5) Complete perioperative records and 30 ± 2 days of follow-up data available.

Exclusion criteria: (1) Age < 12 months or > 108 months; (2) Preoperative organ dysfunction (hepatic, renal, or cardiovascular) or major comorbidities; (3) Incidental discovery of ID during surgical resection of splenic cysts; (4) Participation in other clinical trials.

**Fig. 1:**
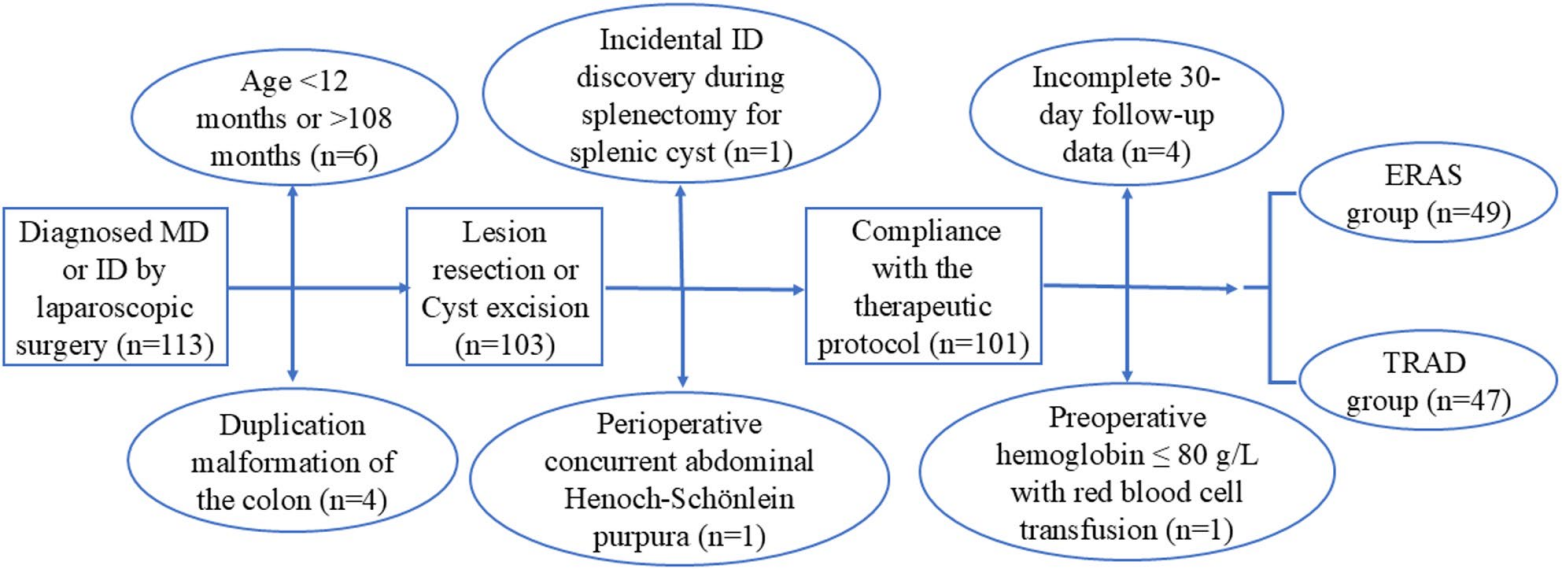
Flowchart of patient selection. Abbreviations: MD = Meckel’s diverticulum; ID = Intestinal duplication; ERAS = Enhanced Recovery After Surgery; TRAD = Traditional care

A total of 96 pediatric patients were included in the analysis, stratified into two groups: the traditional care group (TRAD group, *n* = 47; January 2017–December 2020) and the ERAS protocol group (ERAS group, *n* = 49; January 2021–July 2025). All cases met the inclusion criteria and were performed by the same surgical and anesthesia team. The patient selection process is illustrated in Fig. 1.

This study was approved by the Institutional Review Board of The First Affiliated Hospital of the University of Science and Technology of China (Ethics No: 2025-RE-387). Due to the retrospective observational design and anonymization of data, informed consent from patients or guardians was waived.

### Perioperative management

Surgical timing was determined by pediatric surgeons based on clinical manifestations (abdominal pain, hematochezia, intestinal obstruction, or intussusception) and diagnostic imaging findings (ectopic gastric mucosal scintigraphy, abdominal ultrasonography, or computed tomography [CT] scan). An ERAS multidisciplinary team (MDT) was established, comprising three pediatric surgeons, two anesthesiologists, and five nurses (two operating room nurses and three ward nurses). Adapted from adult ERAS protocols [13], a pediatric-specific protocol was developed to cover all perioperative phases. Preoperatively, families received individualized education on MD/ID management, including dietary guidance, medication instructions, and postoperative care planning; patients in the TRAD group received standard perioperative care. Postoperative pain was assessed using the FLACC (Face, Legs, Activity, Cry, Consolability) scale [14]. Detailed differences in perioperative management between groups are presented in Table 1.

**Table 1:** Comparison of perioperative care measures between the two groups

Category	ERAS group	TRAD group
Preoperation		
1. Patient education	Individualized counseling + structured discharge criteria	Non-mandatory education + standardized instructions
2. Anxiety management	Child-specific interactive media (games/cartoons)	No specific interventions
3. Bowel preparation	Single glycerin enema (morning of surgery)	Dual saline enemas (preoperative night + morning)
4. Preoperative antibiotic	IV cefuroxime sodium (30 min pre-incision)	Identical to ERAS group
5. Fasting protocol	Clear fluids: Clear fluids (10% glucose solution, 5 mL/kg): ≤2 h pre-op; Breast milk: ≤4 h pre-op; Formula/solids: ≤6 h pre-op	Strict NPO for 8 h pre-op
Intraoperation		
6. Tube Management	Selective NG/urinary catheterization: post-induction placement; partial removal in PACU post-awakening	Routine pre-anesthesia placement + prolonged retention
7. Anesthesia technique	General anesthesia + endotracheal intubation + epidural anesthesia, or 0.5% ropivacaine local infiltration	Standard general anesthesia + endotracheal intubation
8. Thermoregulation	Active warming (ambient temperature > 25 °C, forced-air blankets, preheated fluids)	Passive warming only
9. Fluid management	Goal-directed (monitoring of heart rate, blood pressure, urine output; strict Na+/fluid restriction)	Experience-based liberal fluid administration
10. Drain use	Restricted to cases with significant leakage or suspected diverticular perforation	Routine permitted use
Postoperation		
11. Antibiotic prophylaxis	Discontinued within 24 h post-op	Identical to ERAS group
12. NGT	Removed within 48 h post-op (unless output > 50 mL/day of yellow-green fluid)	Retained for 3–4 days post-op (until flatus/defecation)
13. Urinary catheter	Removed within 24 h post-op (low retention rate)	Removed on POD3
14. Abdominal drain	Typically removed within 48 h post-op (once output stabilized)	Removed when drainage < 20mL/day
15. Early mobilization	6 h post-op: Semi-Fowler’s position + assisted bed activities + bedside ambulation (for tolerating patients, especially > 3 years old); POD1: Ambulation for 2 h (for all patients who tolerated 6 h post-op activities)	Bed rest for 2–3 days (no structured protocol)
16. Early oral nutrition	POD1: Clear fluids (water/milk) + gradual diet advancement	Initiated only after flatus/defecation
17. Fluid therapy	IV volume titrated to oral intake	Weight-based standard IV regimen
18. Pain assessment	FLACC scale (2–48 h)	Identical to ERAS
19. Analgesia	Non-pharmacological: Distraction therapy (cartoons/music/oral sucrose); Pharmacological: Scheduled acetaminophen/ibuprofen (q6h if FLACC ≥ 4)	Pharmacological: PRN acetaminophen/ibuprofen (single dose if FLACC ≥ 4)

Footnote: *IV* Intravenous, *NPO* Nil per os, *NGT* Nasogastric tube, *PACU* Post-anesthesia care unit, *POD *Postoperative day, *FLACC* Face, Legs, Activity, Cry, Consolability scale, *PRN* Pro re nata, *q6h* Every 6 hours, *ERAS* Enhanced Recovery After Surgery, *TRAD* Traditional care

The unified standardized pre-discharge assessment items—applied consistently to both the ERAS and TRAD groups—are detailed in Supplementary Table 1. For evaluating perioperative medical and nursing services, respondents who selected “5 = Extremely Satisfied” or “4 = Satisfied” were categorized as “satisfied.” The overall satisfaction rate was calculated as: (number of satisfied respondents / total number of respondents) × 100% [15]. On the 3rd day after discharge, nurses conducted telephone follow-ups to monitor symptoms (e.g., fever, abdominal distension, vomiting) and advised families to attend an outpatient review within 30 ± 2 days postoperatively.

### Surgical procedures

All cases were performed via laparoscopic-assisted approaches, utilizing two standardized techniques: (1) Lesion resection with intestinal anastomosis, or (2) Cyst excision with intestinal repair. The uniform surgical protocol included: (1) Pneumoperitoneum establishment; (2) Comprehensive examination of the proximal small bowel extending to the ligament of Treitz to rule out concurrent ID; (3) Mandatory inspection of ≥ 100 cm of the proximal small intestine to exclude multiple MD; (4) Exteriorization of the affected bowel segment through a 2–3 cm umbilical incision extension. No conversions to open surgery occurred in either group.

Management of MD: (1) Wedge resection for narrow-based diverticula (< 2 cm) with 45° oblique anastomosis; (2) Segmental resection for wide-based diverticula (≥ 2 cm) or complicated cases (diverticulitis/perforation) with end-to-end anastomosis and mesenteric closure; (3) All anastomoses were constructed using continuous 5 − 0 absorbable sutures with interrupted seromuscular reinforcement [16].

Management of ID: (1) For non-communicating cysts: Complete excision with interrupted seromuscular repair; adjacent mucosal injuries were primarily closed with 5 − 0 absorbable sutures followed by seromuscular reinforcement. (2) For communicating cysts: Segmental bowel resection with end-to-end anastomosis and mesenteric repair was performed [17].

Post-anastomotic verification included mucosal integrity inspection, luminal patency testing, and leak exclusion, followed by three-layer umbilical closure (fascial, subcutaneous, cutaneous).

### Discharge criteria and clinical data collection

Discharge was permitted only when all standardized criteria were met: (1) Afebrile with normal urination and defecation; (2) Tolerating oral intake and independent ambulation; (3) Clean, dry surgical incisions (no erythema, swelling, or exudate) and catheter-free; (4) Discontinued intravenous (IV) fluid therapy; (5) No abdominal pain or other complications.

Collected data included: (1) Primary outcomes: Length of stay (LOS) and postoperative complication rate. (2) Secondary outcomes: Perioperative laboratory parameters (including inflammatory markers: white blood cell [WBC] count, C-reactive protein [CRP], neutrophil [NEUT] count; metabolic profiles: glucose, sodium; nutritional indices: hemoglobin [Hb], albumin, prealbumin), postoperative recovery milestones (ambulation, time to first flatus, resumption of oral diet), catheter-related metrics (utilization rate and duration), nutritional support duration (IV nutritional support, total enteral nutrition [TEN]), pain management (FLACC pain scores, epidural catheter metrics), total hospitalization costs, 30-day readmission rate (for complications related to the index surgery), and parental satisfaction. (3) Patient characteristics and intraoperative parameters (preoperative demographics, clinical features, American Society of Anesthesiologists [ASA] classification, malformation type; anesthesia technique, operative type and duration, estimated blood loss, IV fluid administration rate) are retained in a preceding separate paragraph.

### Statistical analysis

All statistical analyses were performed using SPSS 22.0 software (IBM Corp., Armonk, NY, USA). Data distribution was assessed via the Shapiro-Wilk normality test. Parametric continuous variables were expressed as mean ± standard deviation (SD) and compared using Student’s t-test, while non-parametric continuous variables were reported as median (interquartile range [IQR]) and analyzed with the Mann-Whitney U test. Categorical variables were presented as n (%) and compared using the χ² test or Fisher’s exact test (where appropriate). Statistical significance was defined as a two-tailed *P* < 0.05.

## Results

### Preoperative characteristics

A total of 96 pediatric patients were included in the study. The two groups were well-matched in terms of demographic variables (sex, age, weight), clinical features, ASA classification, and malformation type (all *P* > 0.05; Table 2).

**Table 2:** Comparison of demographic and surgical characteristics

Parameter	ERAS group (*n* = 49)	TRAD group (*n* = 47)	*P* value
Gender, n (%)			0.580
- Males	34 (69.39%)	35 (74.47%)	
- Females	15 (30.61%)	12 (25.53%)	
Age (months)	60.00 (36.00, 101.00)	72.00 (31.00, 96.00)	0.730
Weight (kg)	24.00 (16.00, 29.00)	22.00 (14.25, 29.00)	0.547
Clinical features, n (%)			0.761
- Acute onset	16 (32.65%)	13 (27.66%)	0.594
- Chronic onset	29 (59.18%)	31 (65.96%)	0.493
- Asymptomatic	4 (8.16%)	3 (6.38%)	1.000
ASA physical status, n (%)			0.222
- Ⅱ	21 (42.86%)	26 (55.32%)	
- Ⅲ	28 (57.14%)	21 (44.68%)	
Malformation type, n (%)			0.809
- MD	28 (57.14%)	28 (59.57%)	
- ID	21 (42.86%)	19 (40.43%)	
Combined anesthesia, n (%)	41 (83.67%)	13 (27.66%)	< 0.001
Infusion speed (mL/kg/h)	5.37 ± 1.52	7.45 ± 1.69	< 0.001
Catheter utilization, n (%)			
- NGT	19 (38.78%)	31 (65.96%)	0.008
- Urinary catheter	6 (12.25%)	15 (31.92%)	0.020
- Abdominal drain	11 (22.45%)	20 (42.55%)	0.035
Operative duration (min)	93.27 ± 25.13	95.79 ± 25.97	0.630
Estimated blood loss (mL)	10.80 ± 9.38	9.36 ± 7.15	0.403
Surgical technique, n (%)			0.808
- Wr	12 (24.49%)	9 (19.15%)	0.527
- Sr	25 (51.02%)	25 (53.19%)	0.831
- Ce	12 (24.49%)	13 (27.66%)	0.724
Anesthesia recovery time (min)	40.00 (35.00, 45.00)	40.00 (35.00, 45.00)	0.526

Footnote: (1) *ASA* American Society of Anesthesiologists, *MD *Meckel's diverticulum, *ID* Intestinal duplication, *NGT* Nasogastric tube, *Sr* Segmental resection, *Wr* Wedge resection, *Ce* Cyst excision, *ERAS* Enhanced Recovery After Surgery, *TRAD* Traditional care

(2) Data are presented as n (%) for categorical variables, median (25th percentile, 75th percentile) for non-parametric continuous variables, and mean ± standard deviation (SD) for parametric continuous variables

**Fig. 2:**
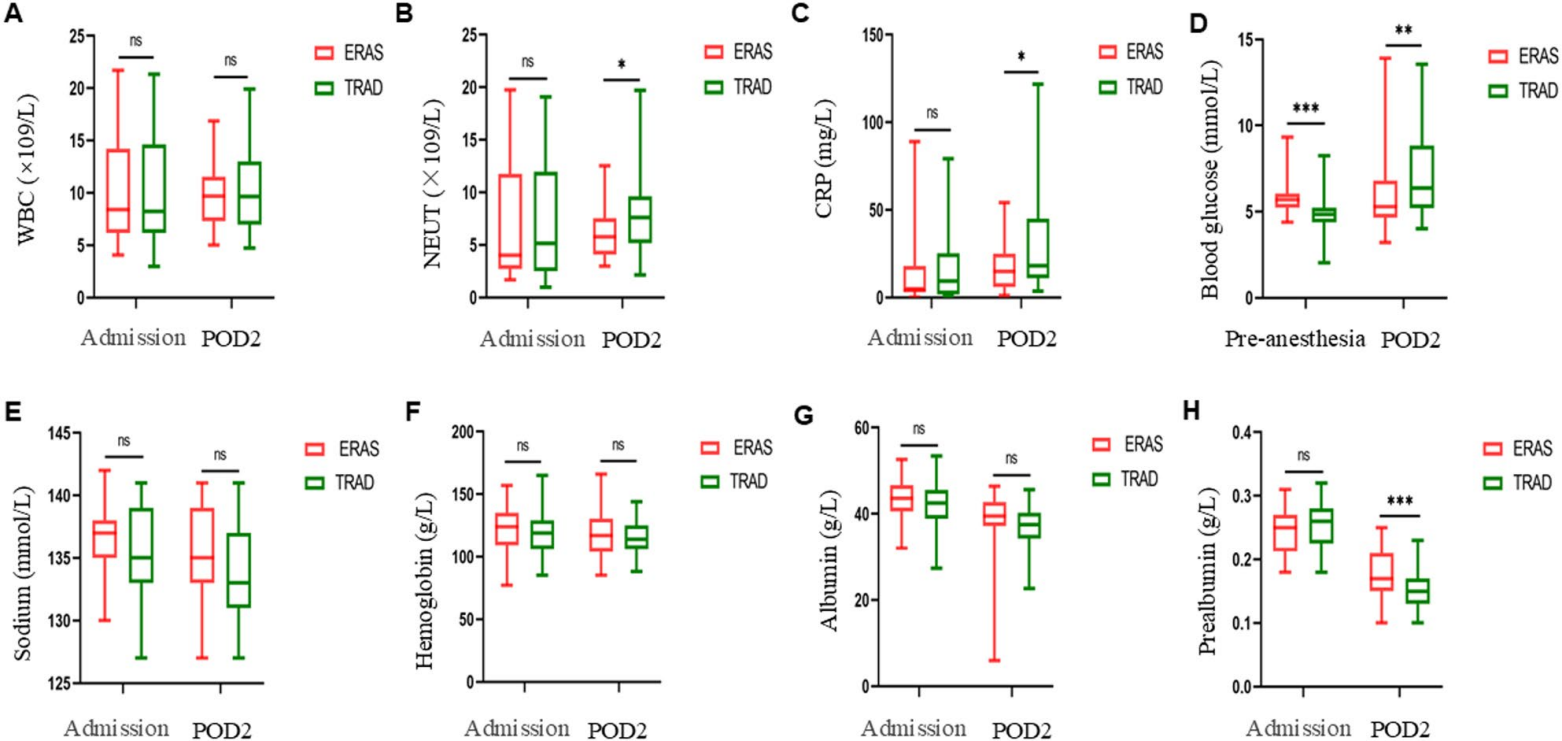
Comparison of perioperative laboratory indicators between the ERAS group (n=49) and TRAD group (n=47) (**A**) White blood cell (WBC) count (×10⁹/L): admission vs. POD2; (**B**) Neutrophil (NEUT) count (×10⁹/L): admission vs. POD2; (**C**) C-reactive protein (CRP) level (mg/L): admission vs. POD2; (**D**) Blood glucose (mmol/L): pre-anesthesia vs. POD2; (**E**) Serum sodium (mmol/L): admission vs. POD2; (**F**) Hemoglobin (g/L): admission vs. POD2;(**G**) Albumin (g/L): admission vs. POD2; (**H**) Prealbumin (g/L): admission vs. POD2. Footnote: (1) Abbreviations: POD = Postoperative day, ERAS = Enhanced Recovery After Surgery; TRAD = Traditional care; (2) Significance: *P<0.05, **P<0.01, ***P<0.001, nsP>0.05 (two-tailed t-test)

Preoperative laboratory findings showed: (1) No significant intergroup differences in inflammatory markers (WBC count, CRP, NEUT count) or nutritional/metabolic indices (Hb, albumin, prealbumin, sodium) (all *P* > 0.05); (2) Significantly higher preoperative glucose levels in the ERAS group compared with the TRAD group (5.70 vs. 4.83 mmol/L, *P* < 0.001). Complete preoperative laboratory data are presented in Fig. 2 and Supplementary Table 2.

### Intraoperative outcomes

All surgical procedures were successfully completed with no mortality. Key intergroup differences in intraoperative outcomes were as follows: (1) Anesthetic management: A higher proportion of pediatric patients in the ERAS group received general anesthesia + endotracheal intubation + epidural anesthesia or 0.5% ropivacaine local infiltration (83.67% vs. 27.66%, *P* < 0.001); (2) Fluid administration: The ERAS group had a lower intraoperative IV fluid infusion rate (5.37 ± 1.52 vs. 7.45 ± 1.69 mL/kg/h, *P* < 0.001) ; (3) Catheter utilization: significantly reduced rates of NGT placement (38.78% vs. 65.96%), urinary catheterization (12.25% vs. 31.92%), and abdominal drainage placement (22.45% vs. 42.55%) in the ERAS group (all *P* < 0.05).

No significant differences were observed between groups in operative duration, estimated blood loss, surgical technique, or anesthesia recovery time (all *P* > 0.05). Complete intraoperative data are detailed in Table 2.

### Postoperative outcomes

Postoperative day 2 (POD2) laboratory analyses showed no significant intergroup differences in WBC count, hemoglobin, albumin, or sodium levels (all *P* > 0.05). However, the ERAS group exhibited favorable metabolic, inflammatory, and nutritional profiles: (1) Nutrition: significantly higher prealbumin levels (0.18 ± 0.04 vs. 0.15 ± 0.03 g/L, *P* < 0.001); (2) Metabolism: lower median glucose (5.30 vs. 6.39 mmol/L, *P* = 0.009); (3) Inflammation: reduced median NEUT count (5.79 vs. 7.60 × 10⁹/L, *P* = 0.030) and CRP levels (14.98 vs. 18.12 mg/L, *P* = 0.042). Complete laboratory data are presented in Fig. 2 and Supplementary Table 2.

To account for potential confounding, a multivariable linear regression model was supplementary performed, adjusted for key covariates (preoperative clinical presentation: acute onset, chronic onset, asymptomatic; age; weight; surgical type; operative duration). As shown in Table 3, after adjusting for potential confounders, the ERAS group was independently associated with significantly lower postoperative CRP levels (β = -16.27, 95% CI: -28.69 to -3.86; *P* = 0.011) and NEUT counts (β = -1.86, 95% CI: -3.43 to -0.28; *P* = 0.021) compared with the TRAD group.

**Table 3:** Multivariable linear regression analysis of the effect of ERAS protocol and confounding factors on postoperative CRP levels and NEUT counts

Variable	Postoperative CRP levels (mg/L)		Postoperative NEUT counts (×10⁹/L)	
	*β* (95% *CI*)	*P*-value	*β* (95% *CI*)	*P*-value
ERAS group vs. TRAD group	-16.27 (-28.69, -3.86)	0.011	-1.86 (-3.43, -0.28)	0.021
Clinical presentation	5.85 (-5.49, 17.19)	0.307	-0.45 (-1.85, 0.96)	0.528
Age	0.05 (-0.34, 0.43)	0.808	0.04 (-0.01, 0.09)	0.110
Body Weight	-0.74 (-2.29, 0.82)	0.348	-0.10 (-0.29, 0.09)	0.314
Surgical type	-6.29 (-16.39, -3.81)	0.218	0.31 (-0.95, 1.56)	0.626
Operative duration	0.22 (-0.03, 0.47)	0.087	0.02 (-0.02, 0.05)	0.313

Footnote: *CRP* C-reactive protein, *β* Unstandardized regression coefficient, *95% CI* 95% confidence interval, *ERAS* Enhanced Recovery After Surgery, *TRAD* Traditional care

The model was adjusted for clinical presentation (acute onset, chronic onset, asymptomatic), age, weight, surgical type (wedge resection, segmental resection, cyst excision), and operative duration as confounding factors. Statistical significance was defined as *P* < 0.05

The ERAS group had significantly shorter median catheter retention durations compared to the TRAD group: NGT (2.00 vs. 4.00 days), urinary catheter (1.50 vs. 2.00 days), and abdominal drain (2.00 vs. 5.00 days) (all *P* < 0.05; Table 4). Epidural catheters were removed immediately postoperatively in infants ≤ 3 years old; in contrast, 6 (12.24%) patients in the ERAS group and 5 (10.64%) in the TRAD group (all ≥ 4 years old) retained epidural catheters for postoperative analgesia. For ERAS group patients not undergoing epidural block, incisional local anesthetic infiltration was routinely administered at the end of surgery. Postoperative analgesia in the ERAS group primarily consisted of intraoperative incisional local anesthesia combined with standardized oral acetaminophen or ibuprofen. The ERAS group achieved superior pain control, with significantly lower mean FLACC scores at 2–48 h postoperatively (all *P* < 0.001; Table 4).

**Table 4:** Comparison of postoperative outcomes

Parameter	ERAS group (*n* = 49)	TRAD group (*n* = 47)	*P* value
Catheter duration (days)			
- NGT	2.00 (2.00, 3.00)	4.00 (3.00, 4.50)	< 0.001
- Urinary catheter	1.50 (1.00, 2.00)	2.00 (2.00, 3.00)	0.014
- Abdominal drain	2.00 (2.00, 3.50)	5.00 (4.00, 5.25)	< 0.001
Pain assessment (FLACC score)			
- 2 h	2.14 ± 0.82	2.96 ± 0.86	< 0.001
- 6 h	3.16 ± 0.85	4.21 ± 0.66	< 0.001
- 12 h	3.74 ± 0.88	5.32 ± 0.86	< 0.001
- 24 h	3.47 ± 0.68	4.21 ± 0.88	< 0.001
- 36 h	2.90 ± 0.68	3.43 ± 0.74	< 0.001
- 48 h	1.84 ± 0.66	2.30 ± 0.66	< 0.001
Epidural catheter management			
- Retained, n (%)	6 (12.25%)	5 (10.64%)	0.805
- Duration (hours)	40.17 ± 6.15	37.20 ± 6.61	0.461
Complications, n (%)	10 (20.41%)	8 (17.02%)	0.671
- Nausea/vomiting	6 (12.25%)	4 (8.51%)	0.791
- Abdominal bloating	3 (6.12%)	2 (4.26%)	1.000
- Cough/sputum production	2 (4.08%)	4 (8.51%)	0.635
- Umbilical infection	2 (4.08%)	0 (0.00)	0.495
- Intra-abdominal infection	1 (2.04%)	2 (4.26%)	0.971
Hospitalization costs (¥)	11181.39 (9732.54, 13402.41)	14102.35 (11812.83, 16741.67)	< 0.001
Parental satisfaction, n (%)	46 (93.88%)	41 (87.23%)	0.444
30-day readmissions, n (%)	1 (2.04%)	2 (4.26%)	0.971
- Incomplete intestinal obstruction	1 (2.04%)	1 (2.13%)	1.000
- Residual abdominal infection	0 (0.00)	1 (2.13%)	0.490

Footnote: (1) *FLACC* Face, Legs, Activity, Cry, Consolability scale, *NGT* Nasogastric tube

(2) Data are presented as n (%) for categorical variables, median (25th percentile, 75th percentile) for non-parametric continuous variables, and mean ± standard deviation (SD) for parametric continuous variables

**Fig. 3:**
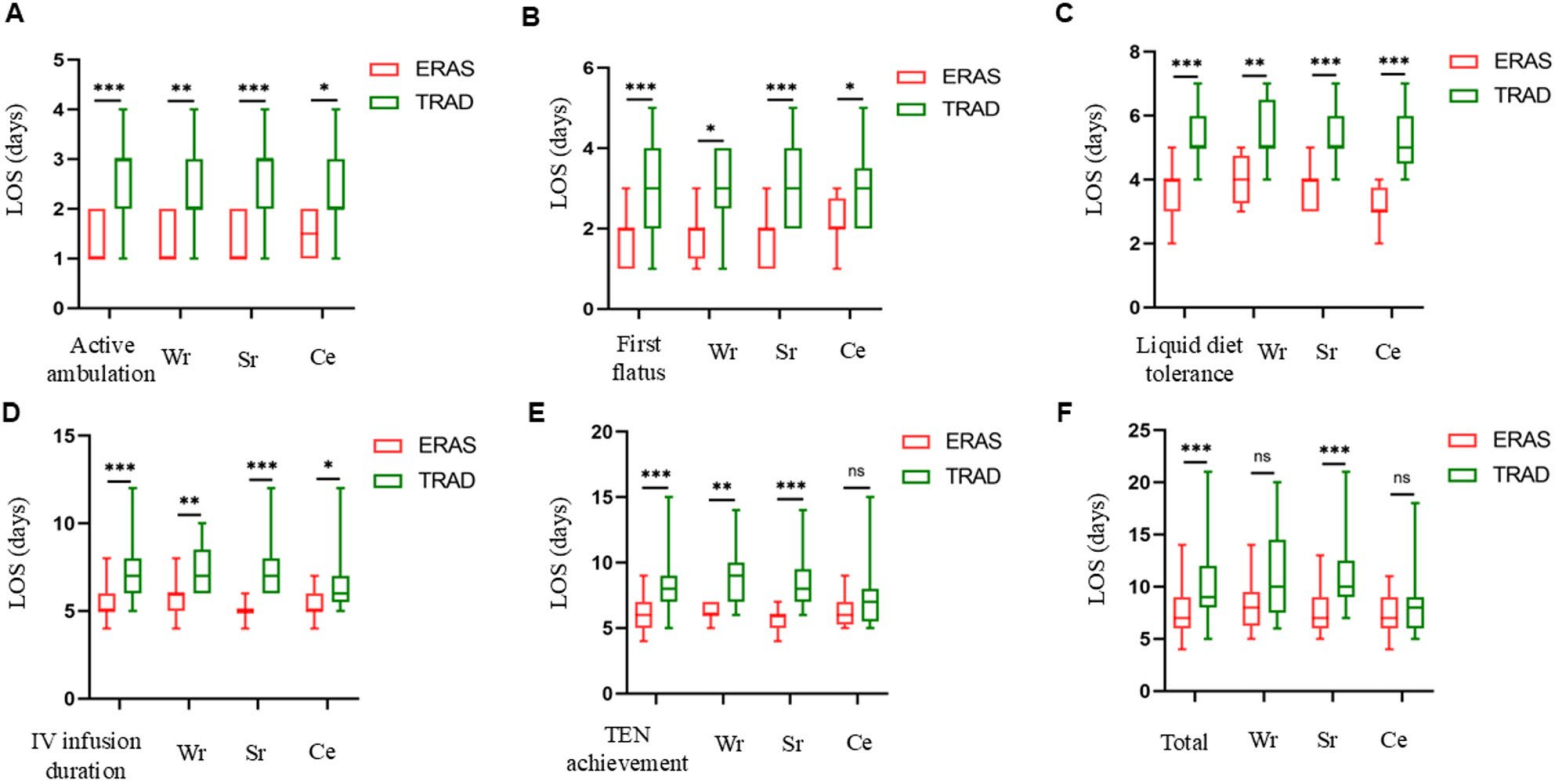
Comparison of postoperative recovery outcomes between the two groups.The ERAS group exhibited superior recovery metrics with significant reductions in: (**A**) Time to active ambulation; (**B**) Time to first flatus; (**C**) Time to tolerance of liquid diet; (**D**) Duration of intravenous (IV) infusion; (**E**) Achievement of total enteral nutrition (TEN);(**F**) Length of stay (LOS) was significantly reduced overall in the ERAS group, with the most pronounced benefit observed in patients who underwent segmental resection and anastomosis for intestinal duplication (ID).Footnote: (1) Abbreviations: TEN = total enteral nutrition, IV = intravenous, LOS = length of stay, Sr = Segmental resection, Wr = Wedge resection, Ce = Cyst excision, ERAS = Enhanced Recovery After Surgery, TRAD = Traditional care; (2) Significance: *P<0.05, **P<0.01, ***P<0.001, nsP>0.05 (two-tailed t-test)

All postoperative recovery milestones were achieved significantly earlier in the ERAS group: active ambulation (1.00 vs. 3.00 days), first flatus (2.00 vs. 3.00 days), liquid diet tolerance (4.00 vs. 5.00 days), discontinuation of IV infusion (5.00 vs. 7.00 days), and achievement of TEN (6.00 vs. 8.00 days) (all *P* < 0.001). These findings confirm the efficacy of ERAS in accelerating gastrointestinal recovery and nutritional restoration (complete data: Fig. 3A–E and Supplementary Table 3).

ERAS significantly reduced median postoperative LOS compared to conventional care (7.00 vs. 9.00 days, *P* < 0.001). Subgroup analysis by surgical procedure revealed notable differences: patients of ID undergoing segmental resection exhibited a more pronounced median LOS reduction in the ERAS group (7.00 vs. 10.00 days, *P* < 0.001), whereas those undergoing cyst excision with repair showed a non-significant trend toward shorter LOS (7.00 vs. 8.00 days, *P* = 0.456). The ERAS protocol demonstrated the strongest efficacy in segmental resection cases (visualized in Fig. 3F).

The ERAS group had a numerically lower complication rate than the TRAD group (20.41% [10/49] vs. 17.02% [8/47], *P* = 0.671). Complication types were similar between groups, including gastrointestinal (nausea/vomiting, bloating), respiratory (productive cough), and local infections (umbilical/intra-abdominal). All complications resolved with conservative management, with no surgical reinterventions required in either group.

The ERAS protocol achieved significant cost savings, with median hospitalization expenses of ¥11,181.39 compared to ¥14,102.35 in the TRAD group (*P* < 0.001). Although parental satisfaction was numerically higher in the ERAS group (93.88% vs. 87.23%), this difference did not reach statistical significance (*P* = 0.444). Thirty-day readmission rates were comparable between groups: 1 case of intestinal obstruction in the ERAS group, and 1 case of intestinal obstruction plus 1 case of abdominal infection in the TRAD group. All readmitted patients resolved completely with conservative treatment, requiring no surgical reintervention (Table 4).

## Discussion

Surgical resection constitutes the definitive curative intervention for MD and ID, given that the vast majority of these lesions are localized to the small bowel [18]. Although laparoscopic surgical approaches have markedly improved clinical outcomes for pediatric patients with these conditions [19], persistent challenges including postoperative morbidity and prolonged hospital LOS [6] underscore the urgent need for optimized perioperative recovery protocols in pediatric gastrointestinal surgery. While robust clinical evidence supports the efficacy of ERAS in adult populations, its translation to pediatric practice remains limited; only 19% of U.S. pediatric surgeons routinely implement ERAS protocols [20], with a notable dearth of standardized guidelines specifically tailored to small intestinal malformations such as MD and ID. To address this critical clinical gap, the present study describes the first comprehensive ERAS protocol designed for laparoscopic-assisted MD/ID resection, demonstrating three key clinical benefits: (1) A significant reduction in median postoperative LOS (shortened by 2 days); (2) Non-inferior safety profiles, as evidenced by comparable complication rates and 30-day readmission rates; and (3) Numerically higher parental satisfaction (93.88% vs. 87.23% in the TRAD cohort).

Pediatric MD and ID are frequently diagnosed only after the onset of acute complications, a diagnostic delay compounded by low baseline parental disease awareness [11]. This clinical reality highlights the critical role of preoperative patient and caregiver education, as well as perioperative counseling, in the success of pediatric ERAS programs. In the present protocol, family engagement was positioned as a central pillar of postoperative rehabilitation, encompassing three core components: (1) Structured support for early mobilization, including assisted ambulation and age-appropriate physical activities; (2) Developmentally tailored comfort measures (e.g., multimedia distraction techniques); and (3) Non-pharmacological interventions to reduce preoperative and perioperative anxiety. This family-centered framework not only accelerated physiological recovery but also enhanced caregiver participation in the rehabilitation process, aligning with core pediatric ERAS principles that prioritize collaborative, family-integrated care.

The unique pediatric surgical stress response—characterized by age-dependent irritability and limited capacity for verbal communication of discomfort [21]—necessitated the adaptation of standard nursing strategies for this cohort. Rather than relying on traditional verbal instructions, interactive play-based interventions were deployed to mitigate preoperative distress in both pediatric patients and their caregivers. This dual focus on family engagement and developmental appropriateness yielded synergistic clinical benefits, reinforcing the principle that pediatric ERAS protocols must balance standardized evidence-based practices with individualized, patient-centric adaptations.

The preoperative fasting regimen in this study adhered to ASA guidelines [22], permitting administration of 10% dextrose solutions until 2 h preoperatively (and 6 h for formula feeds) to maintain metabolic stability while minimizing aspiration risk. This approach delivered three distinct advantages: prevention of preoperative hunger, reduction of caregiver anxiety related to prolonged fasting, and stabilization of perioperative glucose levels. Intraoperatively, the ERAS protocol integrated three core evidence-based components to optimize perioperative physiology: (1) Combined general-epidural anesthesia with 0.5% ropivacaine local infiltration, which minimized opioid exposure and accelerated gastrointestinal motility recovery [23]; (2) Selective placement of NGTs and urinary catheters (post-anesthetic induction) with early postoperative removal, reducing the risk of catheter-associated infections and improving patient comfort; and (3) Goal-directed fluid therapy guided by continuous monitoring of oxygenation, hemodynamic parameters, and central venous pressure (CVP) [24], which mitigated complications of perioperative fluid overload (e.g., hypothermia, cardiopulmonary stress, and intestinal edema) [25]. Collectively, these intraoperative measures ensured perioperative hemodynamic and metabolic stability, laying a foundational framework for accelerated postoperative recovery.

Pain management, widely recognized as the “fifth vital sign” in clinical care [26], serves as a cornerstone of postoperative ERAS protocols. In the ERAS cohort, a standardized 48-hour pain assessment using the FLACC scale was implemented, paired with a multimodal analgesic strategy comprising: (1) Scheduled acetaminophen or ibuprofen; (2) Age-appropriate non-pharmacological interventions (e.g., music or animation with parental participation); and (3) 0.3 mL/kg of 24% sucrose solution for toddlers without nausea or vomiting, leveraging its opioid-mediated analgesic effects [27]. This comprehensive approach resulted in significantly lower postoperative pain scores in the ERAS group compared to the TRAD cohort. However, inherent limitations in pediatric analgesia—including restricted pharmacotherapeutic options for infants and young children—highlight the need for further protocol refinement. Notably, in laparoscopic-assisted pediatric intestinal surgery, the typically small umbilical incision presents an opportunity for targeted regional analgesia; bilateral rectus sheath blocks with dexmedetomidine (providing 24-hour sustained analgesia), combined with IV acetaminophen and pre-oral ketorolac, may not only alleviate postoperative pain and reduce opioid consumption but also contribute to shortened postoperative LOS—a core outcome metric in ERAS implementation.

Selective perioperative tube management represented another critical adaptation of ERAS principles to pediatric MD/ID resection. While NGTs can provide postoperative intestinal decompression to reduce intraluminal pressure and promote anastomotic healing [28], prolonged NGT placement increases the risk of respiratory irritation and pulmonary infection [29]. Consistent with current guidelines discouraging routine NGT use in pediatric distal intestinal procedures [30], the present protocol reserved NGT placement for cases with anatomical or pathological complexity (e.g., duodenal duplication) or preoperative obstruction symptoms, thereby avoiding surgeon-dependent arbitrary utilization. Similarly, urinary catheters were placed only for procedures exceeding 1 h (consistent with the duration of complex ID resections) to enable continuous hemodynamic monitoring, with routine removal within 24 h postoperatively to minimize catheter-related complications. For abdominal drains, placement was restricted to cases with copious turbid peritoneal exudate, balancing the diagnostic benefits (detection of postoperative bleeding, effusion, or fistula) and therapeutic utility (infection prevention). Clinical outcomes validated the value of this tailored approach: only 2 patients in the ERAS cohort required prolonged NGT decompression (≥ 4 days), and 1 patient developed localized abdominopelvic fluid collections—all of which resolved with conservative management and maintained drainage. When combined with optimized analgesia, this modified tube management strategy achieved three key gains: preserved clinical safety, earlier patient mobilization, and accelerated postoperative recovery.

ERAS principles advocate for “graded early ambulation” rather than unrestricted immediate physical activity, and the present protocol implemented a staged rehabilitation model to align with pediatric developmental needs. At 6 h postoperatively, patients-initiated bed-based activities (e.g., semi-Fowler’s positioning, assisted limb movement, and bedside ambulation) to facilitate adaptation to post-surgical physical changes; those who tolerated these activities progressed to full ambulation on POD 1. For patients ≤ 3 years of age (who often exhibit poor ambulation compliance), enhanced parental assistance and nursing supervision were prioritized, while children > 3 years of age initiated active ambulation on POD 1. This individualized approach eliminated fear associated with forced activity, improved patient and caregiver acceptance, and aligned with pediatric ERAS principles of progressive, developmentally appropriate rehabilitation.

Early EN was a critical component of the protocol, offering three key physiological benefits: attenuation of postoperative hypermetabolism, stabilization of stress-induced glucose fluctuations, and promotion of anabolic recovery [31]. Traditional pediatric surgical protocols mandate prolonged fasting until the return of bowel function (defined by flatus or defecation) to prevent gastrointestinal complications, but emerging evidence indicates that prolonged postoperative fasting impairs wound healing, induces immunosuppression, and increases infection risk [32]. The nutritional strategy in the present study included controlled hydration and carbohydrate supplementation starting on POD 1, with gradual advancement to full EN—without a corresponding increase in gastrointestinal complications (e.g., abdominal distension or emesis). Metabolic optimization was confirmed by POD 2 laboratory findings: the ERAS cohort exhibited lower median glucose levels, higher prealbumin concentrations (a sensitive marker of acute nutritional status), and maintained serum sodium levels (indicating no hemodilution from excessive fluid administration). This approach balanced the benefits of early feeding with clinical safety, validating the role of early EN in mitigating postoperative catabolism and enhancing recovery in pediatric patients [33].

Biochemically, the ERAS protocol demonstrated efficacy in modulating the pediatric surgical stress response. NEUT count—a key mediator of the inflammatory cascade—and CRP—a hepatocyte-derived acute-phase protein—are known to peak 24–48 h postoperatively in pediatric intestinal surgery, reflecting the degree of surgical trauma and systemic inflammation [34, 35]. POD 2 laboratory analysis in the present study revealed significantly lower CRP levels and NEUT counts in the ERAS group (both *P* < 0.05), confirming that multimodal ERAS interventions (e.g., early EN, minimally invasive anesthesia, and optimized fluid management) effectively mitigate perioperative inflammation. These findings provide objective biochemical validation of ERAS’s capacity to preserve postoperative homeostasis while accelerating clinical recovery in pediatric MD/ID resection.

Subgroup analysis by surgical procedure type revealed differential ERAS efficacy across MD/ID subtypes: patients undergoing segmental intestinal resection experienced a more pronounced reduction in median LOS (7.00 vs. 10.00 days, *P* < 0.001), whereas those undergoing cyst excision with repair exhibited a non-significant trend toward shorter median LOS (7.00 vs. 8.00 days, *P* = 0.456). This discrepancy likely reflects the greater surgical trauma associated with segmental resection (e.g., prolonged intestinal manipulation and the physiological demands of anastomotic healing), highlighting the need for procedure-specific ERAS adaptations in future protocol iterations.

Several limitations of the present study warrant careful consideration. First, the generalizability of inflammatory marker findings may be constrained, as POD 2 assessment of CRP, WBC count, and NEUT count aligns with institutional clinical pathways but is not universally adopted in pediatric MD/ID resection. Second, the reported median LOS (7 days in the ERAS cohort; 9 days in the TRAD cohort) exceeds that of large national datasets, including the National Surgical Quality Improvement Program-Pediatric (NSQIP-Pediatric, 4 days for open MD surgery) [36] and other published series [37, 38]. This discrepancy is attributable to three key factors: (1) The study cohort included both MD and ID (with ID associated with greater anatomical complexity and delayed gastrointestinal recovery) and a younger age range (1–8 years), which confers higher risks of postoperative feeding intolerance; (2) Conservative institutional discharge criteria requiring 2–3 days of observation after IV fluid discontinuation to confirm tolerance of TEN; and (3) variable initial protocol adherence (e.g., timing of mobilization and discharge planning), which may have blunted the magnitude of LOS reduction. Third, the retrospective study design limits causal inference, though multivariable regression adjusting for key confounders (age, weight, clinical presentation, surgical type, and operative duration) strengthens the validity of the observed ERAS-associated outcomes.

Despite these limitations, the present study fills an important gap in the pediatric surgical literature by validating ERAS efficacy in a combined MD/ID cohort with an extended age range, complementing existing evidence focused on isolated pediatric MD or broader pediatric gastrointestinal surgery [39, 40]. To further optimize postoperative outcomes in this high-risk population, four targeted refinements to the pediatric ERAS protocol are proposed: (1) Preoperative enteral nutrition support: Eligible patients will receive 3–7 days of high-calorie formula supplementation preoperatively to enhance anastomotic healing potential and accelerate postoperative gastrointestinal function recovery. (2) Pediatric-specific prokinetic interventions: Starting on POD 1, erythromycin (for infants) and standardized chewing gum stimulation (for children > 3 years of age) will be incorporated to promote bowel function recovery—a key determinant of LOS in patients undergoing intestinal anastomosis. (3) Objective discharge readiness metrics: Fixed observation periods will be replaced with standardized, validated criteria (e.g., feeding tolerance scores and daily gastrointestinal function assessments) to enable earlier, safe discharge of eligible patients (e.g., older children with uncomplicated MD). (4) Participation in pediatric ERAS consortia: Collaboration with specialized pediatric ERAS consortia will facilitate outcome benchmarking, adoption of evidence-based best practices, and validation of the optimized protocol in larger, geographically diverse pediatric cohorts.

In conclusion, the tailored ERAS protocol described herein safely optimizes postoperative recovery in pediatric patients undergoing laparoscopic-assisted MD/ID resection, with significant benefits observed in LOS, pain control, metabolic stability, and healthcare cost reduction. While current LOS remains higher than benchmark data, targeted protocol refinements and multicenter randomized controlled trials—focused on age-stratified optimization and long-term outcomes—will further enhance its clinical utility. These findings support the broader adoption of ERAS in pediatric small bowel malformation surgery, with actionable insights to improve care quality and outcomes for high-risk pediatric surgical populations.

## Supplementary Information

Supplementary Material 1

Supplementary Material 2

Supplementary Material 3

## Data Availability

All data supporting the findings of this study are available from the corresponding author(s) upon reasonable request.
