# Supplementary material for: Advancing enhanced recovery after surgery protocols for pediatric laparoscopic-assisted small intestinal malformation repair
Source: BMC Pediatr. 2026 Jan 20;26:338. doi: 10.1186/s12887-026-06516-z (PMC13088440; doi:10.1186/s12887-026-06516-z)
Supplement: Supplementary file 1 — Supplementary Material 1 [file 12887_2026_6516_MOESM1_ESM.docx]

Supplementary Table 1. Unified discharge pre-survey items

| **Parameter** | **Likert Scale: 1 -5** |
| --- | --- |
| **Preoperative Management (Consistent for Both Groups)** |  |
| - Preoperative health education (clarity of disease explanation, perioperative preparation guidance, protocol introduction) |  |
| - Communication with medical staff (diagnostic clarity, surgical plan/risk explanation, responsiveness to parental queries) |  |
| - Psychological support for children and parents (efficacy in alleviating preoperative anxiety) |  |
| **Intraoperative & Postoperative Care (Consistent for Both Groups)** |  |
| - Timeliness and clarity of intraoperative progress feedback to parents |  |
| - Postoperative vital sign monitoring (nursing carefulness and professionalism) |  |
| - Wound care and infection prevention (dressing changes, wound healing guidance) |  |
| - Ward environmental comfort (hygiene, noise control, facility convenience) |  |
| **Core Perioperative Interventions (Observable Before Discharge)** |  |
| - Pain management (analgesic efficacy, pain assessment frequency, symptom relief) |  |
| - - ERAS group: Multimodal analgesia + regular pain assessment + individualized adjustment |  |
| - - TRAD group: Symptom-based traditional analgesia (e.g., as-needed medications) |  |
| - Early mobilization (clarity of activity guidance, nursing support, child’s activity tolerance) |  |
| - - ERAS group: Protocol-based guided early ambulation (≤24 h postoperatively) |  |
| - - TRAD group: Traditional activity recommendations (e.g., bed rest until wound pain relief) |  |
| - Nutritional support (postoperative feeding timing/type, oral intake guidance, child’s acceptance) |  |
| - - ERAS group: ERAS principle-based early oral feeding (e.g., clear liquids 12–36 h postoperatively) |  |
| - - TRAD group: Traditional fasting protocol (e.g., NPO until return of bowel sounds) |  |
| - Gastrointestinal function recovery (guidance on defecation/flatus, management of nausea/vomiting, observed recovery progress before discharge) |  |
| **Discharge Preparation (Consistent for Both Groups)** |  |
| - Discharge guidance (clarity of medication use, wound care, home activity/diet advice) |  |
| - Completeness of discharge materials (follow-up appointment details, medical records, care reminders) |  |
| - Recognition of the child’s recovery progress before discharge |  |
| **Overall Perception (Consistent for Both Groups)** |  |
| - Overall satisfaction with perioperative medical and nursing services |  |
| - Willingness to recommend the hospital/department to others (relatives/friends) |  |

**Footnote**: (1) Likert scale (1 = Extremely Dissatisfied, 5 = Extremely Satisfied). (2) Abbreviations: NPO = Nil Per Os; ERAS = Enhanced Recovery After Surgery; TRAD = Traditional care.
