# Supplementary material for: Advancing enhanced recovery after surgery protocols for pediatric laparoscopic-assisted small intestinal malformation repair
Source: BMC Pediatr. 2026 Jan 20;26:338. doi: 10.1186/s12887-026-06516-z (PMC13088440; doi:10.1186/s12887-026-06516-z)
Supplement: Supplementary file 2 — Supplementary Material 2 [file 12887_2026_6516_MOESM2_ESM.docx]

Supplementary Table 2. Comparison of perioperative laboratory indicators

| **Parameter** | **ERAS group (n=49)** | **TRAD group (n=47)** | ***P value*** |
| --- | --- | --- | --- |
| **WBC counts (×10^9^/L)** |  |  |  |
| - Admission | 10.13±4.84 | 10.02±5.01 | 0.915 |
| - POD 2 | 9.58±2.78 | 10.20±4.23 | 0.457 |
| **NEUT counts (×10^9^/L)** |  |  |  |
| - Admission | 4.04 (2.74, 11.71) | 5.16 (2.60, 11.84) | 0.947 |
| - POD 2 | 5.79 (4.14, 7.34) | 7.60 (5.20, 9.60) | 0.030 |
| **CRP (mg/L)** |  |  |  |
| - Admission | 4.15 (3.73, 14.93) | 9.41 (1.95, 24.47) | 0.943 |
| - POD 2 | 14.98 (6.08, 24.26) | 18.12 (11.28, 43.80) | 0.042 |
| **Blood glucose (****mmol/L)** |  |  |  |
| - Pre-anesthesia | 5.70 (5.25, 6.05) | 4.83 (4.41, 5.19) | <0.001 |
| - POD 2 | 5.30 (4.67, 6.72) | 6.39 (5.21, 8.19) | 0.009 |
| **Sodium (mmol/L)** |  |  |  |
| - Admission | 136.43±2.84 | 135.06±3.79 | 0.097 |
| - POD 2 | 135.27±3.82 | 134.04±3.92 | 0.125 |
| **Hemoglobin (g/L)** |  |  |  |
| - Admission | 121.02±18.97 | 118.00±16.58 | 0.411 |
| - POD 2 | 116.36±17.76 | 114.89±12.96 | 0.686 |
| **Albumin (g/L)** |  |  |  |
| - Admission | 43.57±4.68 | 41.91±5.31 | 0.107 |
| - POD 2 | 38.82±7.21 | 36.42±5.56 | 0.129 |
| **Prealbumin (g/L)** |  |  |  |
| - Admission | 0.24±0.04 | 0.25±0.04 | 0.429 |
| - POD 2 | 0.18±0.04 | 0.15±0.03 | <0.001 |

**Footnote**: (1) WBC = white blood cell; NEUT = neutrophil; CRP = C-reactive protein; POD = postoperative day; ERAS = Enhanced Recovery After Surgery; TRAD = Traditional care. (2) Data are presented as median (25th percentile, 75th percentile) for non-parametric continuous variables, and mean ± standard deviation (SD) for parametric continuous variables.
