# Supplementary material for: Advancing enhanced recovery after surgery protocols for pediatric laparoscopic-assisted small intestinal malformation repair
Source: BMC Pediatr. 2026 Jan 20;26:338. doi: 10.1186/s12887-026-06516-z (PMC13088440; doi:10.1186/s12887-026-06516-z)
Supplement: Supplementary file 3 — Supplementary Material 3 [file 12887_2026_6516_MOESM3_ESM.docx]

Supplementary Table 3. Comparison of postoperative recovery indicators

| **Parameter** | **ERAS group (n=49)** | **TRAD group (n=47)** | ***P value*** |
| --- | --- | --- | --- |
| **Active ambulation (days)** | 1.00 (1.00, 2.00) | 3.00 (2.00, 3.00) | <0.001 |
| - Wr | 1.00 (1.00, 2.00) | 3.00 (2.00, 3.00) | 0.004 |
| - Sr | 1.00 (1.00, 2.00) | 3.00 (2.00, 3.00) | <0.001 |
| - Ce | 1.50 (1.00, 2.00) | 2.00 (2.00, 3.00) | 0.01 |
| **First flatus (days)** | 2.00 (1.00, 2.00) | 3.00 (2.00, 4.00) | <0.001 |
| - Wr | 2.00 (1.75, 2.00) | 3.00 (3.00, 4.00) | 0.011 |
| - Sr | 2.00 (1.00, 2.00) | 3.00 (2.00, 4.00) | <0.001 |
| - Ce | 2.00 (2.00, 2.25) | 3.00 (2.00, 3.00) | 0.016 |
| **Liquid diet tolerance (days)** | 4.00 (3.00, 4.00) | 5.00 (5.00, 6.00) | <0.001 |
| - Wr | 4.00 (3.75, 4.25) | 5.00 (5.00, 6.00) | 0.003 |
| - Sr | 4.00 (3.00, 4.00) | 5.00 (5.00, 6.00) | <0.001 |
| - Ce | 3.00 (3.00, 3.25) | 5.00 (5.00, 6.00) | <0.001 |
| **IV infusion duration (days)** | 5.00 (5.00, 6.00) | 7.00 (6.00, 8.00) | <0.001 |
| - Wr | 6.00 (5.00, 6.00) | 7.00 (6.00, 8.00) | 0.004 |
| - Sr | 5.00 (5.00, 5.00) | 7.00 (6.00, 8.00) | <0.001 |
| - Ce | 5.00 (5.00, 6.00) | 6.00 (6.00, 7.00) | 0.039 |
| **Time to achieve TEN (days)** | 6.00 (5.00, 7.00) | 8.00 (7.00, 9.00) | <0.001 |
| - Wr | 6.00 (6.00, 7.00) | 9.00 (7.00, 9.00) | 0.003 |
| - Sr | 6.00 (5.00, 6.00) | 8.00 (7.00, 9.00) | <0.001 |
| - Ce | 6.00 (5.75, 7.00) | 7.00 (6.00, 7.00) | 0.260 |
| **LOS (days)** | 7.00 (6.00, 9.00) | 9.00 (8.00,11.50) | <0.001 |
| - Wr | 8.00 (6.75, 8.50) | 10.00 (8.00, 12.00) | 0.090 |
| - Sr | 7.00 (6.00, 9.00) | 10.00 (9.00, 12.00) | <0.001 |
| - Ce | 7.00 (6.00, 9.00) | 8.00 (6.00, 9.00) | 0.456 |

**Footnote**: (1) TEN = total enteral nutrition; IV = intravenous; LOS = length of stay; Sr = Segmental resection; Wr = Wedge resection; Ce = Cyst excision. (2) Data are presented as median (25th percentile, 75th percentile).
